# 本草物质科学研究方法与技术进展

**DOI:** 10.3724/SP.J.1123.2026.03008

**Published:** 2026-08-08

**Authors:** Yanfang LIU, Han ZHOU, Jixia WANG, Aijin SHEN, Dian LIU, Yang HAN, Wenyi YU, Bin YUAN, Zhen WANG, Jie ZENG, Xingya XUE, Jiatao FENG, Zhimou GUO, Xinmiao LIANG

**Affiliations:** 1. 中国科学院大连化学物理研究所，植物化学与天然药物全国重点实验室，辽宁 大连 116023; 1. State Key Laboratory of Phytochemistry and Natural Medicines，Dalian Institute of Chemical Physics，Chinese Academy of Sciences，Dalian 116023，China; 2. 赣江中药创新中心，江西 南昌 330000; 2. Ganjiang Chinese Medicine Innovation Center，Nanchang 330000，China; 3. 中国科学院上海药物研究所，上海 201203; 3. Shanghai Institute of Materia Medica，Chinese Academy of Sciences，Shanghai 201203，China

**Keywords:** 本草物质组, 中药现代化, 多维制备色谱, 靶点发现, 人工智能, 结构生物学, herbalome, modernization of traditional Chinese medicine, multidimensional preparative chromatography, target discovery, artificial intelligence, structural biology

## Abstract

中药作为复杂的物质体系，长期面临“物质基础不明确、作用机理不清晰”的挑战，制约其现代化与国际化发展。为解决这一问题，“本草物质组计划”于2007年提出，旨在通过多学科技术整合，系统解析中药的物质组成、结构及其生物学功能，阐明多成分-多靶点协同作用机制。本文综述了本草物质科学研究的关键方法与技术进展：在本草物质组成与结构解析方面，液相色谱-质谱联用技术结合二维色谱、智能数据挖掘策略，实现了中药复杂化学成分的高通量表征与新化合物发现；多维高效制备色谱技术及自主研制的多维多通道分离纯化装置，突破了中药化合物规模化、系统化制备的瓶颈；核磁共振技术结合人工智能（如HSQC智能识别、深度学习模型），显著提升了结构鉴定的效率与精度。在本草物质的生物效应研究方面，靶点导向与表型导向的药理学技术（如无标记细胞整合药理学技术、热蛋白质组分析技术、亲和质谱技术）助力中药活性成分的靶点发现与机制解析；中药数据库与计算模拟技术（如虚拟筛选、分子动力学、人工智能预测模型）支撑了“多组分-多靶点”复杂互作网络的构建与模拟；结构生物学技术（尤其是冷冻电镜）为中药活性成分与靶点蛋白的相互作用提供了原子水平的结构洞察，推动了构效关系与多靶点机制的阐释。综上，分离分析、生物效应评价与计算模拟等多种技术的协同发展，正推动本草物质科学研究从“经验依赖”向“数据驱动”的范式转变。新技术的不断涌现与融合将持续揭示中药的未知领域，系统阐明其多成分多靶点的协同作用机制，深度发掘新结构、新靶点及新生物学效应，为中药传承创新和现代新药研发提供源头创新动力。

## Abstract (en)

Traditional Chinese medicine （TCM）， as a complex system of substances， has long faced the challenges of an unclear material basis and obscure mechanisms of action， which has hindered its modernization and internationalization. To address these challenges， the “Herbalome” project was launched in 2007. This initiative aims to systematically reveal the material composition， structure， and biological functions of TCM through the integration of multidisciplinary technologies， thereby elucidating the synergistic mechanisms of multi-component and multi-target interactions. This paper reviews key methodological and technological advancements in the field of herbal material science. Specifically， regarding the composition and structural identification of herbal compounds， the integration of liquid chromatography-mass spectrometry with two-dimensional chromatography and intelligent data mining strategies has enabled high-throughput characterization of the complex chemical constituents of TCMs， as well as the discovery of novel compounds. Furthermore， advancements in multidimensional high-performance preparative chromatography and the development of novel multidimensional multi-channel separation and purification devices have overcome bottlenecks in the large-scale systematic preparation of TCM compounds. Additionally， nuclear magnetic resonance technology， enhanced by artificial intelligence techniques， such as intelligent heteronuclear single quantum coherence （HSQC） recognition and deep learning models， has significantly improved the efficiency and accuracy of structural identification. In the study of the biological effects of herbal substances， target-oriented and phenotype-oriented pharmacological technologies， such as cellular label-free integrative pharmacology， thermal proteome profiling， and affinity mass spectrometry， have facilitated the discovery of targets and the elucidation of mechanisms for bioactive components in TCM. Moreover， TCM databases and computational simulation techniques， including virtual screening， molecular dynamics， and artificial intelligence prediction models， have supported the construction and simulation of complex “multi-component and multi-target” interaction networks. Structural biology techniques， particularly cryo-electron microscopy， have provided atomic-level insights into the interactions between TCM bioactive components and target proteins， advancing our understanding of structure-activity relationships and multi-target mechanisms. In conclusion， the synergistic advancement of various techniques， including separation analysis， bioeffect evaluation， and computational modelling， is driving a paradigm shift in herbal material science from “experience-dependent” to “data-driven” approaches. The ongoing emergence and integration of novel technologies will continue to reveal unexplored areas within traditional Chinese medicine， systematically elucidate its multi-component， multi-target synergistic mechanisms， and enable the in-depth discovery of novel structures， targets， and biological effects. This progress will provide innovation insights to support the inheritance and innovation of traditional Chinese medicine as well as modern drug discovery.

中医药是中国古代科学的瑰宝，为中华民族的繁衍昌盛和人类健康做出了不可磨灭的贡献。中医药的传承创新发展既是人民群众的医疗健康和国家公共卫生的迫切需求，也是我国医药创新的重大需求。中药是一个高度复杂的物质体系，有着长期的临床实践和大量的人用经验。由于研究方法和技术的局限性，中药研究长期面临“物质基础不明确，作用机理不清晰”的根本问题，难以从科学层面阐明中药多成分多靶点的协同作用机制，无法回答中药有效性/安全性的科学原理，这严重制约了中药传承创新发展。

2007年10月28日，梁鑫淼团队在东方科技论坛“本草物质组计划”学术研讨会上首次提出“本草物质组计划”，旨在全面解析中药物质组成、结构和功能，构建本草物质资源库，阐述中药的多组分多靶点整体调节机制。该计划为我国中药现代化科学研究描绘出较为清晰的路线图，具有十分重要的科学意义和深远影响^［1］^。2008年，Science以“揭开中药神秘面纱”为题报道了“本草物质组”计划^［2］^，同年，香山科学会议举行了以“本草物质组”为主题的第323次学术讨论会，与会专家一致认为本草物质组以中医药传承创新发展为宗旨，遵循源于传统、高于传统的原则，以中药研究的标准化、现代化和国际化为目标，力求在现代科学技术发展的基础上，通过技术与方法的创新与突破，阐明中药药效物质基础，回答中药治疗疾病的内在科学原理。

在多项国家项目的支持下，梁鑫淼团队突破了中药物质组成、结构和功能系统解析的关键技术，如正交性分离材料^［3-5］^、多维高效制备色谱方法^［6］^、无标记整合药理学技术^［7-10］^等，为本草物质组研究奠定了扎实的基础。同时也逐步认识到当前的仪器装备落后，严重制约了本草物质组的研究效率，亟需构建一个物质组成解析、作用靶点筛选和协同机理研究的一体化研究设施，提供极限的研究手段去探明这个未知世界，发现中药科学原理。2019年6月和2023年10月香山科学会议分别举行了以“本草物质科学研究设施及应用”和“本草物质科学与临床医学”为主题的第S50次和第731次学术讨论会，两次会议的与会专家对梁鑫淼团队建议的研究设施的科学性、重要性、紧迫性以及可行性进行充分论证，认为建设本草物质科学研究设施十分必要。

2023年12月，中国科学院大连化学物理研究所圆满完成了本草物质科学研究设施的预研工作，构建了世界上第一套多维多通道分离纯化预研装置，该装置坐落于江西省赣江新区^［11］^。该预研装置大幅提升了中药靶效物质的研究效率和研究深度，发现了大量新骨架、新结构以及新生物学功能，充分体现了中药这一资源宝库的价值和本草物质科学研究的深远意义。

本草物质科学研究是一个需要融合多学科交叉技术攻关的大科学命题，近年来，多组学技术、人工智能（AI）技术、冷冻电镜（Cryo-EM）等新技术的快速发展，有力推动了中药科学原理向分子层面的深入。本文综述了本草物质组成结构和生物效应研究方法和技术进展，旨在为本草物质科学领域的研究提供参考。

## 1 本草物质组研究总体目标、研究路线及技术方案

本草物质组研究的总体目标是以中药研究的标准化、资源化、现代化和国际化为目标，继承中医药理论和临床实践，系统深入地开展本草物质组的研究，以方法学的创新与突破为重点，发展高通量超高效的制备和表征技术与平台，全面解析本草物质基础、创建本草物质、信息和知识资源库；以资源为导向，重点开展本草物质高内涵筛选和系统生物学的研究，阐述中药的物质基础、作用原理及其多组分多靶点整合调控机制，解读中药的科学性，丰富和发展中医药理论；建立完整的知识产权保护体系，为持续、高效的重大创新中药研究提供技术与资源支撑，为中药现代化与国际化做出积极贡献^［1］^。

围绕上述总体目标制定的研究路线如图1所示，中药复方由多个饮片组成，通过标准化的分离纯化，将饮片提取物分离成一个个组分，再从组分制备出单体化合物进行结构鉴定，明确中药物质组成和结构；针对这些化合物通过高通量活性筛选发现活性化合物，由活性化合物回归到活性组分，由多个靶点活性组分进行组合，阐明协同作用机制，最终形成中药新药。

**图1:**
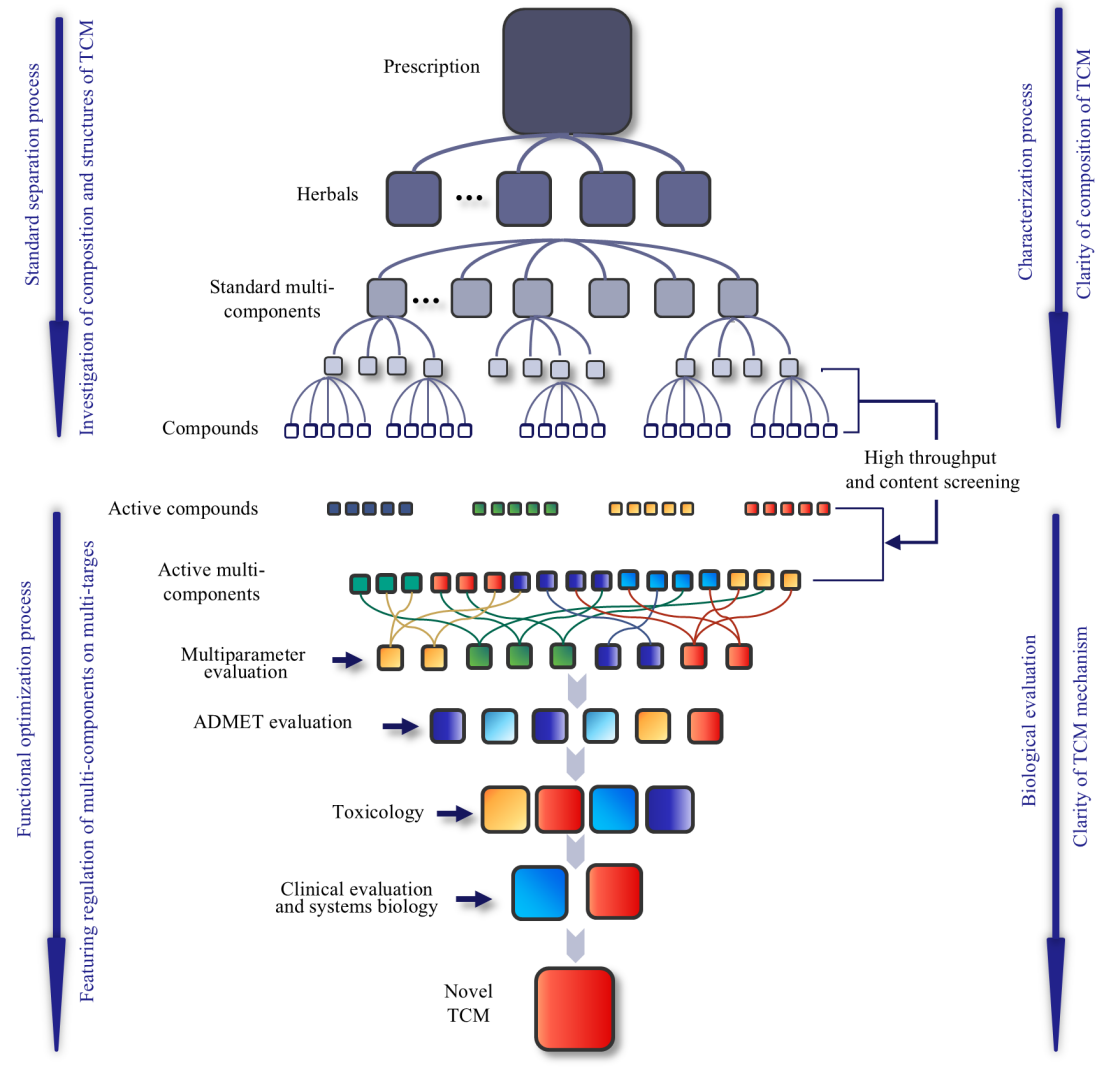
本草物质研究路线

该研究路线的落地实施，亟需两大核心研究领域的技术突破：一是突破本草物质组成结构研究方法和技术，发展规模化、标准化、高通量的分离纯化技术，快速获得中药化合物样品，发展AI驱动的液相色谱-质谱联用和核磁表征技术，提升化合物结构鉴定效率；二是突破生物效应研究方法和技术，发展高通量靶点发现、活性分子与靶蛋白的分子作用机制研究等先进生物学技术，精准揭示活性成分的作用靶点、调控机制及药理效应。二者互为支撑、有机耦合，是本草物质科学研究的核心关键。

## 2 本草物质组成与结构研究方法与技术

本草物质组成与结构研究旨在利用现代分离纯化和结构解析技术全面、系统地阐明本草物质的物质基础，构建覆盖本草物质化学成分组成、结构特征的“本草物质组”，为揭示中药药效物质基础、阐释作用机制及实现精准质量控制提供关键科学依据。本草物质的分离纯化是全面解析本草物质组成和结构的基础。中药是一个典型的复杂物质体系，涵盖多种类型化合物，其理化性质与成分含量差异显著，同分异构体普遍存在，其分离纯化与结构解析面临诸多挑战：质谱解析数据庞杂，人工解析模式效率不足；传统分离纯化技术效率低下，难以实现化合物的系统制备；传统核磁共振（NMR）技术受限于低灵敏度和高度专家依赖性，难以满足高通量与高精度研究需求。

近年来，该领域取得了一系列重大突破：液相色谱-质谱联用技术结合二维色谱与分子网络、数据库匹配、基于机器学习算法的智能解析等数据处理策略，显著提升了中药复杂成分的高通量表征与新化合物发现能力；多维高效制备色谱技术的发展与多维多通道分离纯化预研装置的成功研制，大幅提升了本草物质分离纯化的规模化、通量和研究深度；AI结构解析技术通过融合深度学习与多源光谱数据已实现从复杂谱图到分子结构端到端的精准预测与自动生成，极大地推动了高通量、高精度的结构鉴定进程。

### 2.1 液相色谱-质谱联用分析

中药物质组成的系统解析是阐明中药物质基础的核心前提，也是揭示其作用机理的必要基础。然而，中药化学成分极为复杂，单味药往往包含数百至上千种化合物，这些化合物在结构特征、极性分布及含量丰度上差异显著，给系统解析与精确鉴定带来了巨大挑战。液相色谱-高分辨串联质谱联用技术（LC-HRMS/MS）凭借其高选择性、高灵敏度和多维信息获取等独特优势，已成为中药全成分表征和新化合物发现的关键技术手段^［12］^。

在色谱分离方面，高效液相色谱（HPLC）或超高效液相色谱（UHPLC）可以实现复杂中药样品的快速分离，特别是近年来快速发展的正交二维色谱分离技术，包括离线二维和在线二维两种模式，可以显著提升色谱分离的峰容量和选择性，有效减少不同成分在质谱离子化过程中的相互干扰，从而增强对中药样品中微量乃至痕量成分的检测能力。本研究团队将正交二维色谱技术成功应用于中药方剂或饮片中不同结构类型成分的系统分离，并结合质谱技术进行表征。如采用反相×反相离线二维色谱分离清肺排毒汤，获得了749个色谱峰，鉴定了其中498个成分，为清肺排毒汤治疗新冠肺炎的作用机制研究提供了化学成分基础^［13］^；采用正相×反相离线二维色谱分离甘遂提取物，结合质谱鉴定了其中218个二萜类成分，其中包括141个未报道的巨大戟烷型和麻风树烷型二萜，为先导化合物的发现提供了新的目标^［14］^；采用反相×超临界流体离线二维色谱分离雷公藤，鉴定了其中324个成分，包括284个生物碱、22个二萜和18个三萜，极大丰富了雷公藤的物质基础信息^［15］^；构建了不同pH体系的反相×反相在线二维正交体系，从钩吻中发现并鉴定了256个吲哚型生物碱，其中包括156个未报道的成分^［16］^。在线二维分离技术大大提高了中药复杂体系分离的通量与自动化程度，克服了离线二维分离技术操作烦琐、耗时长且重复性相对差等问题，但在线二维体系的构建相对复杂，且对第一维与第二维流动相体系的兼容性要求高、灵活性相对差。因此，根据中药化学成分类型选择合适的分离技术至关重要。

在质谱解析方面，LC-HRMS/MS在一次实验中往往会产生成千上万张质谱图，传统人工解析模式效率低下，如何在庞杂的数据中高效提取目标信息，并实现成分的精准表征与新化合物的快速挖掘，已成为液相色谱-质谱联用技术表征中的一大难点。为应对这一挑战，研究者们发展出多种数据采集与处理策略（图2）。代表性方法有中性丢失扫描（neutral loss scanning， NLS）、质量亏损过滤（mass defect filtering， MDF）、中性丢失过滤（neutral loss filtering， NLF）、诊断/关键离子过滤（diagnostic/key ion filtering， DIF/KIF）和分子网络/特征分子网络（molecular networking/feature-based molecular networking， MN/FBMN）等。这些方法充分利用同类型化合物在质谱裂解中呈现的共有特征，对复杂中药样品的质谱数据进行高效筛选和过滤。例如，MDF与KIF分别借助质量亏损规律与骨架特征碎片来识别目标分子；NLF和NLS则依赖含有相同结构片段的化合物在碎裂过程中表现出的相同质量丢失，以实现目标结构类型的靶向采集或过滤；MN/FBMN则利用质谱图的相似性，将结构相关的化合物进行聚类，从而加速已知骨架的确认和新类型化合物的发现。通过综合选择多种扫描或过滤方法，可以实现对数据的筛选、降维和聚类，不仅大幅降低了数据处理的复杂性，还显著提升了针对特定类别化学成分的发现效率。本研究团队结合NLS和MDF对射干的化学成分进行分析，快速筛选并鉴定了65个异黄酮苷元（42个未报道）和142个异黄酮糖苷（122个未报道）^［17］^；结合FBMN与多级质谱裂解，从苦参中鉴定了186个喹诺里西啶类生物碱（131个未报道）^［18］^；利用KIF结合Python算法构建了MS-SMART智能策略，从地不容、黄连、黄柏、关黄柏、夏天无五种富含异喹啉类生物碱的药材中成功发现并鉴定了422个新的小檗碱类成分^［19］^。

**图2:**
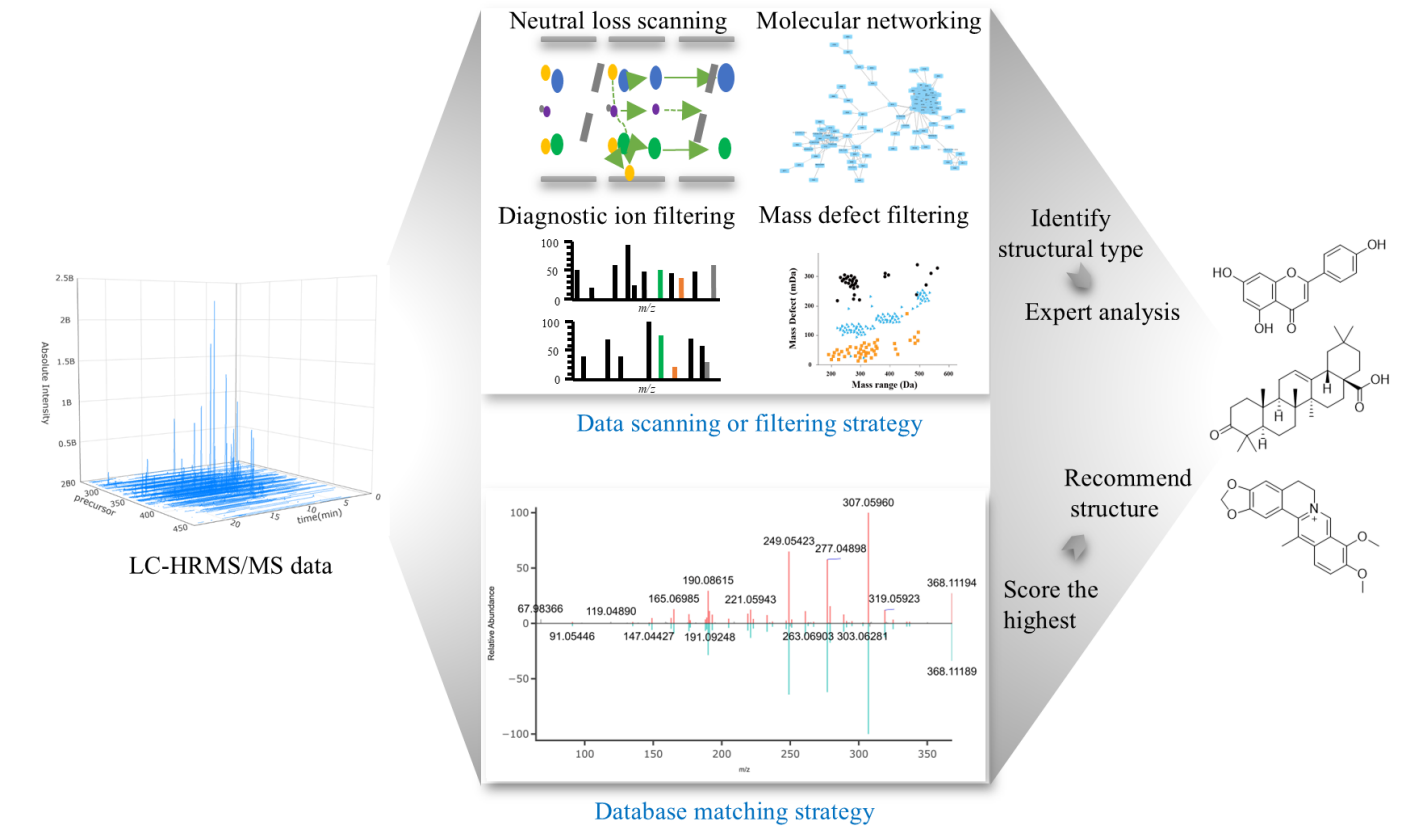
中药化合物液相色谱-高分辨串联质谱数据经典处理策略

除了上述技术之外，数据库匹配（database matching， DM）也是一种重要的质谱数据快速处理策略，通过与已有的标准质谱图或参考数据库进行比对，实现化学成分的快速鉴定。该方法具有通用性强、效率高等优势，已成为中药成分研究中常用的数据解析手段之一^［20］^。然而，现有质谱数据库在覆盖度上仍存在明显不足，其所收录的化合物数量远低于中药复杂体系中实际存在的成分数量与多样性。这一局限性不仅制约了数据库匹配在中药全成分解析中的应用深度，也凸显了构建更大规模、更高质量且具有中药特色的质谱数据库的迫切需求。随着人工智能技术的迅速发展，基于机器学习算法的质谱智能解析逐渐成为研究热点。通过引入深度学习、迁移学习等方法，实现对中药化合物理论谱图的快速预测，或基于实验获得的目标图谱推测其结构类别及潜在结构。Sebastian Böcker团队利用深度神经网络构建了从质谱碎片预测化合物类别的机器学习模型CANOPUS^［21］^，成功实现不同大戟属植物化学成分的结构类型预测，加速新天然产物的快速发现。这一策略不仅有望显著降低对现有标准质谱库的依赖，还能在一定程度上弥补数据库覆盖不足的局限性。同时，机器学习模型能够在处理大规模复杂数据时展现出更高的效率和准确性，从而大幅提升中药复杂体系中化学成分的解析与新化合物的发现能力。

### 2.2 多维高效制备液相色谱技术与装置

分离纯化技术是阐明本草物质结构的必要手段，利用分离纯化技术获得本草物质，是阐明其生物功能的关键基础。本草物质组成复杂，涵盖数千种小分子，包括生物碱、黄酮、苯丙素、萜类和有机酸等多种类型的化合物，其理化性质和含量丰度差异显著，且普遍存在同分异构体与手性对映体，对分离纯化技术提出极高要求。当前，中药分离纯化技术平台以柱层析技术为主，中压制备色谱和高效制备色谱技术为辅，分离能力和制备效率低下，远不能满足中药化合物系统制备的需求，基于上述技术构建的中药化合物库数量有限，只能提供单味饮片的个别化合物，无法提供能够代表饮片或方剂整体的化合物样品，严重阻碍了中药药效物质基础和作用机理研究，是中药有效性、安全性的科学阐释一直未能突破的“卡脖子”问题之一。

研究发现色谱固定相的粒径越小，柱效越高，分离能力也越强^［22］^，因此，色谱固定相逐步向小粒径方向发展。采用高压泵，柱效更高，并加快了液相色谱的分离速度。相比于柱层析技术，高效制备色谱技术具有高效、快速、自动化程度高、重复性好、分离能力强、可在线检测等优势^［23-25］^。与中压制备色谱技术相比，高效制备液相色谱技术在柱效上提高了1个数量级，峰容量显著增加^［26］^。然而中药样品由于其复杂性，即使采用高效制备液相色谱技术也无法一次制备出所有化合物，因此，一维色谱常常受峰容量和分离度的限制，难以满足中药化合物系统全面分离的需求。

近年来，出现了制备型在线二维液相色谱，样品经过第一维的色谱柱进入接口，通过浓缩、富集或切割后进入第二维液相色谱中进行分离，实际峰容量较一维制备色谱提高5倍。如德国的Sepiatec公司推出的SEPBOX 2D-2000二维高通量分离制备色谱系统，国内苏州汇通公司也推出了类似的仪器。理论表明，在充足的分析时间内，二维色谱的峰容量将比一维色谱高出一个数量级，但在实际的二维色谱分离中，样品峰的分布并不是像理想状态下的有序分布，而是呈现无序分布的状态。因此，即使实际的二维色谱峰容量有很大提高，以至于能够完全容纳所有组分的谱带宽度，但因样品峰的无序分布，仍不能完全满足复杂样品的分离要求。因此，二维制备色谱技术在一定程度上提高了中药化合物制备的覆盖度，但由于中药化合物的无序分布，尤其是大量异构体的存在，仍然不能满足中药物质基础全面解析的需求。

多维高效液相制备色谱技术是在高效制备液相色谱的基础上，通过在线或离线再进样操作，结合多种分离模式或分离机理而形成的一项能对复杂样品实现高容量分离纯化的新技术，可实现样品不同组分在一次操作中的多次分离。它不仅能提高色谱体系的峰容量和正交性，还能降低色谱峰之间的重叠，使得中药中化合物的全面获取成为可能。但在实际应用中存在3个难点问题（图3），一是多维色谱的峰容量取决于相邻两维之间的正交度，而固定相对正交度的影响往往是决定性的，因此多维高效制备需要发展新型色谱分离材料，以实现相邻两维之间较高的正交度，从而使多维色谱的峰容量呈指数级增长；二是随着分离维度增加，样品质量逐步减少，分离维度越大需要的药材总量越大，制备规模也越大，同时也使得整个分离时间成倍增加，因此，需要构建规模化、高通量、自动化的分离纯化装置，实现中药化合物的多维高效制备，获得mg级至g级样品以供本草物质结构和功能的深入研究，尤其是突破微量新骨架化合物的制备；三是中药化合物结构多样，且每种中药成分不同，因此如何针对每种中药的每一维制备选择柱系统，构建多维高效制备色谱方法也是需要解决的难题。

**图3:**
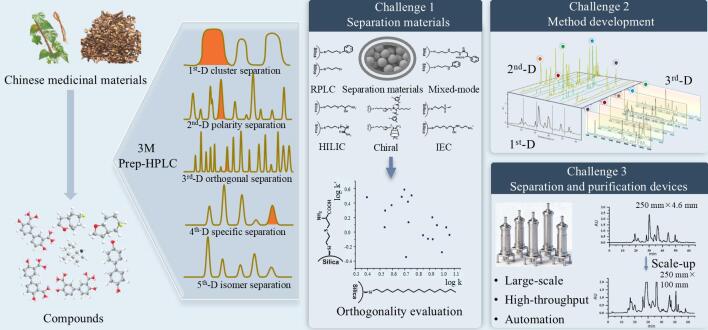
本草物质多维高效液相制备色谱技术

#### 2.2.1 新型色谱分离材料研发进展

近年来，针对本草物质复杂样品的特性，研究者们开发了一系列具有特定分离机制和优异性能的新型固定相材料。硅胶基质固定相因具有高机械强度、可控的粒径/孔径结构和比表面积，以及较好的化学稳定性和热稳定性、专一的表面化学反应，是目前应用最为广泛的一类无机填料^［27］^，是本草物质分离纯化的基础。硅胶表面富含硅羟基，可通过硅烷化技术键合各种官能团，构建多种色谱模式，包括反相液相色谱（reversed-phase liquid chromatography， RPLC）、正相液相色谱（normal-phase liquid chromatography， NPLC）和亲水相互作用液相色谱（hydrophilic interaction liquid chromatography， HILIC）。硅胶基质固定相主要集中在表面改性，提高对不同化学成分的保留和选择性，以适应复杂基质中不同类型化合物的高效分离^［28］^。表面改性主要通过硅烷化反应在硅胶表面接枝功能基团，形成键合相。如烷基硅烷化通过疏水相互作用用于反相分离；氨基、硫酸基等用于离子交换固定相；五氟苯基固定相（PFP）通过其*π-π*相互作用和偶极-偶极作用用于黄酮类等芳香类化合物的选择分离^［29］^。本研究团队开发了一系列针对本草物质分离的固定相材料，Song等^［3］^开发了一种含硫醚、氨基和羟基的新型含氮杂环键合相（AMTA），在皂苷、黄酮及生物碱分离中表现出良好的正交性和峰形；Song等^［4］^开发了一种全氟辛烷键合硅胶固定相（FC8），利用其对夏天无生物碱进行分离纯化时，表现出优于传统PFP固定相的分离性能；Ding等^［5］^开发的苯基氨丙基键合硅胶（PHN）表面电荷稳定、载样量高，在低pH条件下对生物碱的分离效果优于商品化CSH Phenyl-Hexyl固定相，已成功应用于延胡索中去氢延胡索乙素的纯化。

#### 2.2.2 分离纯化装置研发进展

在中国科学院和江西省的大力支持下，梁鑫淼团队突破系列核心部件设计加工和性能验证，研制了国际原创的多维多通道分离纯化预研装置^［11］^。该装置坐落于江西省赣江新区，是一套五维平行制备色谱，包括一维聚类分离、二维极性分离、三维正交分离、四维特异性分离和五维异构体分离5个分离单元，每个单元均为多个通道平行运行。目前该装置已稳定运行2年，大幅提升了本草物质分离纯化的规模化、高通量和自动化，为本草物质科学的深入探索以及创新药物的发现，提供了至关重要的物质基础与源头保障。

#### 2.2.3 多维高效制备色谱方法进展

随着制备维度的增加，不同维度的色谱柱系统的选择缺乏方法学研究指导，采用常规的试错法，构建效率较低，费时费力。本研究团队针对多维高效制备色谱系统构建问题，提出了多维多模式多柱（multi-dimension multi-mode multi-column， 3M）制备色谱策略^［6］^。发展了邻二维色谱柱快速选择^［30］^、多维色谱柱系统推荐^［6］^、制备条件快速优化、大尺度模拟制备等方法技术，形成了标准化流程，有效提升了多维高效制备色谱方法开发效率。目前基于3M策略构建的多维高效制备色谱系统已成功用于北豆根、钩藤、刺山柑等中药的分离纯化，例如：从北豆根中制备鉴定了136个化合物，其中77个为新化合物^［6，31-33］^。该结果凸显了多维高效制备色谱技术强大的分离能力。融合先进分离材料、多维多通道分离纯化装置和多维高效制备液相色谱方法学，该技术将显著提升本草化合物制备效率和研究深度，为本草物质结构与功能研究提供根本保障。

### 2.3 核磁鉴定方法

结构鉴定作为本草物质科学的核心任务，旨在系统阐明中药及复方中数以万计的化学成分，全面绘制“本草物质组”，从而为揭示中药药效物质基础、阐释作用机制及实现精准质量控制提供关键科学依据。现代结构鉴定技术常常联用紫外（UV）、红外（IR）、质谱（MS）和核磁共振四大光谱进行表征。具体而言，紫外与红外光谱可提供关键的官能团信息，质谱以其极高的灵敏度和质量精度可用于快速筛查和分子式推测，而核磁共振则能够提供原子间的连接方式、相对构型及空间构象等精细结构信息，成为结构确证的“金标准”。然而，传统核磁共振技术受限于低灵敏度导致的数据采集时间长，以及对复杂谱图解析的高度专家依赖性，难以满足大规模研究的需要。

为应对本草物质科学对高通量、高精度结构解析的双重需求，一系列创新核磁鉴定技术相继涌现，从不同层面推动此类瓶颈问题的突破，核磁共振技术在结构解析中的发展如图4所示。中药复杂体系中含有大量已知化合物，因此亟需实现快速、精准的结构鉴定与高效去重复化。现有专业数据库（如微谱、ACD/Structure Elucidator）虽在一定程度上实现了计算机辅助识别，但仍严重依赖^13^C NMR数据，存在灵敏度低、样本需求量高和数据库覆盖有限等问题，导致大量已知物仍需人工比对，严重制约了鉴定效率。近年来，HSQC智能识别技术的兴起有效突破了传统碳谱方法的局限。该技术不仅灵敏度显著优于^13^C NMR，所需样品量更少，而且每个C-H相关信号具有独特的分子“指纹”特性，抗干扰能力强，尤其适合中药复杂体系中微量成分的高通量筛查。袁斌等^［34］^开发的HSQCid算法采用对比学习框架，结合图神经网络与卷积神经网络，通过HSQC/^13^C谱图与结构的自监督学习实现跨模态识别，在无约束实验数据中Top 1准确率达74.5%，Top 10达94.8%，超越了传统碳谱基本检索方法。Kim等^［35］^提出的DeepSAT系统，仅利用HSQC谱图即可识别相关已知化合物，显著提升了分子结构的鉴定效率。这类技术通过高效利用HSQC谱图中丰富的相关信号，实现了中药已知化合物的秒级精准鉴定，极大地推动了高通量、高精度结构鉴定体系的发展。

**图4:**
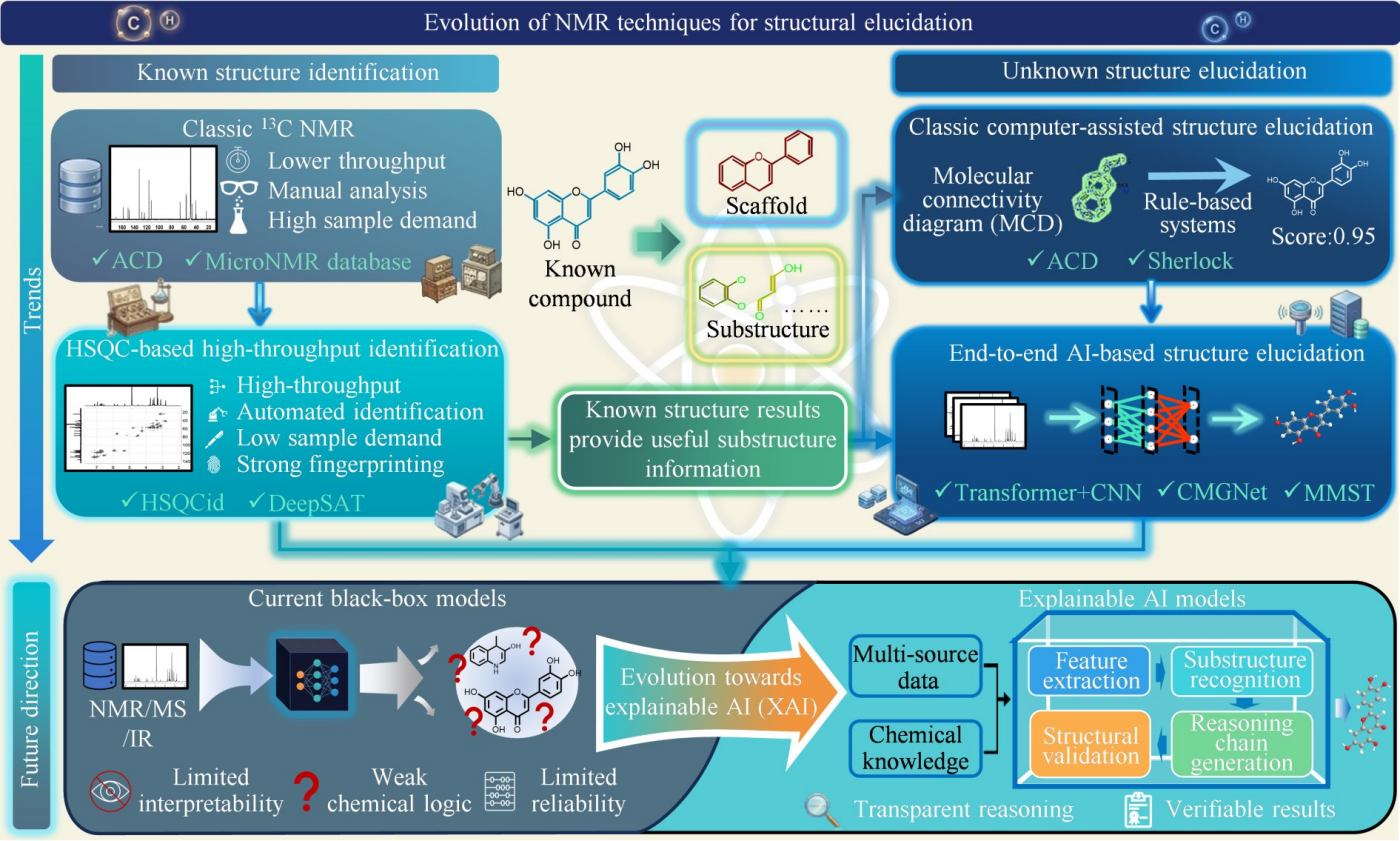
核磁共振技术在结构解析中的发展

在实现已知化合物高效鉴定的基础上，中药复杂体系中大量未知成分的结构解析仍是核心挑战。为提高未知成分的解析效率，计算机辅助结构解析（CASE）系统（如ACD/Structure Elucidator^［36］^、Sherlock^［37］^）通过融合算法推理与谱图数据库，为结构鉴定提供了重要支持。然而，此类系统在高通量、高精度解析方面仍存在明显局限性：其基于规则和模板匹配的推理机制难以适应中药体系的复杂性，枚举策略导致处理效率低下；对谱图重叠和低信噪比数据的解析精度不足；且缺乏自主学习能力，需依赖大量人工干预，严重制约了其实际应用。近年来，人工智能技术尤其是深度学习的引入，为中药未知成分的结构解析开辟了新路径。Hu等^［38］^构建了基于Transformer与卷积神经网络的端到端模型，仅凭一维NMR谱即可高效预测含19个重原子以内的分子结构，无需先验知识即可将搜索空间缩减11个数量级，Top15预测准确率达69.6%。Yao等^［39］^提出的条件分子生成网络（CMGNet），融合^13^C NMR、分子式与片段信息，在Top10预测中实现94.17%的准确率，在多类化合物生成与结构修正中表现优异。Priessner等^［40］^开发的多模态Transformer（MMST）模型整合多种谱学数据（MS、1D/2D NMR及IR等），依托注意力机制进行端到端预测，仅经模拟数据训练即对真实样本达到94%的准确率，显著推进了自动化结构解析进程。这些AI结构解析技术通过自主学习化合物与核磁谱图间的复杂映射规律，实现了化学位移的精确预测与候选结构的智能生成，显著降低了解析不确定性及对专家经验的依赖，有效弥补了传统CASE系统的局限性，为未知成分的高通量、高精度结构鉴定提供了关键技术支撑。

尽管现有智能结构预测算法可快速生成候选结构，但仍普遍缺乏可解释性与明确的化学逻辑支撑，限制了其在本草物质科学中的深入应用。因此，发展可解释性人工智能模型，增强算法透明度和可靠性，促进AI与化学认知的深度融合，将成为下一代智能结构解析工具的发展方向，推动中药结构研究从“黑箱预测”迈向“可解释、可验证”的推理新范式。

## 3 本草物质生物效应研究方法与技术

本草物质生物效应研究旨在利用现代生物学技术阐释多成分多靶点作用机制，其本质上是解决传统中药研究中“药效物质不清、作用机制模糊”的核心痛点，破解中药“哪些化合物通过哪些靶点起效”黑箱效应，是推动传统中药向现代创新药物转化及深化疾病机制认识的关键。然而，中药是一个复杂物质体系，其“多成分-多靶点-多功能”的特性使得从分子水平揭示其多靶点作用机制面临巨大挑战。

近年来，随着化学生物学、结构生物学及人工智能等前沿学科的发展，该领域取得了重大进展：无标记细胞整合药理学技术、热蛋白质组分析技术、亲和质谱技术等靶点导向与表型导向的药理学技术助力中药活性成分的靶点发现与机制解析；中药数据库与虚拟筛选、分子动力学、人工智能预测模型等计算模拟技术支撑了“多组分-多靶点”复杂互作网络的构建与模拟；以冷冻电子显微镜为代表的结构生物学技术为中药活性成分与靶点蛋白的相互作用提供了原子水平的结构洞察，推动了构效关系与多靶点机制的阐释。

### 3.1 靶点活性发现技术

靶点是药物与机体生物大分子的结合部位，主要包括受体如G蛋白偶联受体（GPCR）、酶、离子通道、转运体和核酸等。药物通常通过作用于特定靶点并调控其生物学效应，从而实现疾病治疗作用。中药作用靶点发现是中药现代化、精准化及国际化发展的核心环节。然而，中药是一个复杂物质体系，如何从分子水平揭示其多靶点机制，已成为中药研究领域的重大挑战，也是本草物质科学的重要研究内容。近年来，随着分子生物学、化学生物学、生物物理学等前沿学科的发展，研究者建立了中药作用靶点发现的系列技术方法，主要分为反向药理学技术（即靶点导向的活性发现技术）和正向药理学技术（即表型导向的靶点解析技术）两大类，这两类技术在中药靶点活性发现中取得了重要进展。

靶点导向的活性发现技术是以特定疾病相关的靶点（如GPCR、酶、核受体、离子通道）为目标，在分子水平（如酶、受体）或细胞水平（如受体转染、报告基因细胞系）建立模型，通过生物物理分析等技术手段，从中药化合物中识别出能够与之结合并调节其功能的活性化合物，其最大的特点是可直接得到中药化合物的作用靶点。代表性技术主要包括高通量实时荧光检测技术（fluorescent imaging plate reader， FLIPR）、无标记细胞整合药理学技术（cellular label-free integrative pharmacology， CLIP）、亲和质谱技术（affinity mass spectrometry， AMS）及表面等离子共振技术（surface plasmon resonance， SPR）等。FLIPR是一种基于荧光信号实时监测的高通量细胞功能分析技术，常用于检测细胞内的GPCR第二信使钙流变化或膜电位变化，其原理是通过荧光染料标记细胞，当化合物与靶点结合后，可引起细胞内钙浓度或电生理特性的改变，从而被荧光读板机实时捕获^［41］^。该技术高通量、高灵敏度，适用于大规模化合物库筛选，但是需使用荧光染料，可能存在染料毒性或干扰，对某些靶点（如非钙依赖通路）适用性有限。该技术已成功应用于中药靶点活性化合物的发现，如从荷叶中发现*O-*去甲荷叶碱、*S*-乌药碱、*R*-乌药碱能够拮抗多巴胺D1和D2受体^［42］^。为了解决传统检测技术的荧光染料标记干扰及在研究GPCR等细胞信号转导中通路信息局限的问题，基于细胞表型的CLIP技术逐步发展起来，其原理是当外界刺激如药物或化合物与细胞表面或内部的靶点结合后，会引起细胞形态、黏附性或细胞骨架的重排，细胞内效应物质位置会改变而发生动态质量重置（dynamic mass redistribution， DMR），基于折射率是细胞底部（~150 nm）物质浓度的函数，细胞内这种质量再分布会引起照射在生物传感器底部的偏振光发生位移，被记录为可视的DMR信号谱^［43，44］^。该技术无需标记，可实时监测细胞整体响应，能够反映靶点及下游信号通路的整合效应，但需要监测整个动力学过程，检测时间较长，且信号解析复杂。本研究团队将该技术成功用于中药GPCR靶点活性发现及通路解析，如从镇痛中药钩吻中发现双靶点活性化合物二氢钩吻素子，既部分激动阿片μ受体MOR，又能拮抗毒蕈碱M3受体^［7］^，在急性和炎症性疼痛模型中均能有效缓解疼痛，且无阿片类药物常见的耐药副作用，揭示了钩吻临床镇痛的双靶点机制，也为长期疼痛管理镇痛剂的开发奠定了基础；从新疆紫草中发现紫草素激活GPR120受体，并解析了下游通路信号，揭示了紫草素降糖的分子机制^［8］^；同时利用CLIP技术解析了方剂的多成分多靶点机制，如清肺排毒汤抗新冠肺炎^［9］^及复方金钱草抗前列腺炎^［10］^的多靶点机制。为了解决如何从复杂体系快速发现靶点活性化合物的问题，基于亲和选择与质谱检测结合的亲和质谱技术随之发展起来，其原理是将靶点蛋白固定于载体或溶液中，与化合物孵育，随后通过洗脱或直接进样，利用质谱的高灵敏度鉴定与靶点结合的化合物^［45］^。该技术灵敏度高，适用于中药复杂样品的筛选，但无法区分活性属性且数据分析具有较大挑战。基于该技术，水雯箐教授研究团队成功从粉防己提取物中发现了血清素受体5-HT2C的靶点活性化合物^［46］^。以上技术在中药靶点化合物发现方面具有较好的优势，但受限于化合物的可获得性及筛选模型的有限性，发现的靶点活性化合物较为零散且随机性大，难以应对靶点系统发现。

为了解决目标化合物（通常具有药效表型）靶点系统发现的问题，表型导向的靶点解析技术逐步发展起来，该类技术通常以某一化合物为目标，将标记/非标记化合物与生物样本（细胞裂解液、动物组织匀浆等）共孵育，结合靶点钩钓、热蛋白组分析等，鉴定化合物的作用靶点。代表性技术主要包括基于亲和探针的靶点鉴定技术（如生物素标记、光亲和标记及点击化学的炔基/叠氮基标记）和基于蛋白稳定性的靶点鉴定技术如热蛋白质组分析技术（thermal proteome profiling， TPP）等。基于生物素标记的靶点鉴定技术是国际上靶点鉴定的重要方法^［47，48］^，其原理是将生物素（biotin）通过连接臂与小分子化合物偶联，形成生物素标记探针，探针与靶点结合后，利用生物素与链霉亲和素的高亲和力，通过链霉亲和素富集，鉴定靶点。该技术在复杂的蛋白组中能捕获出低丰度蛋白靶点，但探针设计复杂，修饰基团可能会影响小分子与靶点的结合，需通过结构优化减少干扰。利用该技术，屠鹏飞/曾克武教授研究团队鉴定了野马追中野马追内酯B的作用靶点，发现其作用于去泛素化酶USP7靶点，来抑制小胶质细胞的激活，从而达到抗神经炎症的作用^［49］^。为了解决探针合成困难及修饰影响活性的问题，非标记靶点发现技术因其不改变分子结构且普适性强的优点而被发展和应用。TPP是一种将细胞热位移分析（cellular thermal shift assay， CETSA）与质谱技术相结合的非标记靶点发现技术，其原理是当化合物与目标蛋白发生结合时，蛋白质构象、分子内疏水性等可能会发生改变，从而影响蛋白的热稳定性，通过对比加药组与对照组蛋白质热熔曲线的变化，即可确定是否发生了靶点结合^［50］^。该技术一次实验可同时分析数千种蛋白质，适用于挖掘作用机制不明确化合物的作用靶点，但该技术对蛋白类型有局限性，主要适用于可溶性蛋白，对膜蛋白检测困难，后续靶点确认的工作量大。该技术被成功应用于化合物的靶点鉴定，如庾石山教授研究团队发现了有毒天然产物羟基马桑毒素通过激活钙调磷酸酶靶点诱发癫痫发作，揭示了癫痫的新发病机制^［51］^；Dziekan等^［52］^鉴定了嘌呤核苷磷酸化酶为抗疟药物奎宁的作用靶点，揭示了该药物抗疟的新机制。表型导向的靶点解析技术在鉴定目标化合物靶点中发挥了重要作用，但由于非特异性靶点干扰，需结合靶点导向活性发现技术验证作用靶点的可靠性；此外，还需考虑共孵育生物样本中蛋白靶点表达情况，以达到相应效应相关靶点的发现。各项中药作用靶点发现技术的优势与不足如表1所示。

**表1:** 中药作用靶点发现技术的优势与不足

Representative technology	Advantages	Disadvantages
Fluorescent imaging plate reader	high throughput， high sensitivity， suitable for large-scale compound library screening	requiring fluorescent dyes， potential dye toxicity or interference， limited applicability to certain targets （e.g.， non-calcium-dependent pathways）
Cellular label-free integrative pharmacology	label-free， real-time monitoring， integrated response of targets and pathways， high throughput	long detection time， complex signal analysis
Affinity mass spectrometry	high sensitivity， suitable for screening traditional Chinese medicine	inability to distinguish active properties， significant challenges in data analysis
Biotin-based target identification	enabling capture of low-abundance protein targets， precise localization	difficulty in probe design and synthesis， modifying groups may affect the binding of the compound to the target
Thermal proteome profiling	no modification required， thousands of proteins analyzed simultaneously in one experiment	limited protein types， complex data processing， difficulty in subsequent target validation

### 3.2 中药数据库与计算模拟技术

中药的“多成分-多靶点-多功能”的特点生成了海量的物质组成数据和生物医学数据，因此“本草物质组”计划的一个重要目标是构建本草物质数据库，形成覆盖“物质组成解析—药物靶点发现—生物效应验证—疾病机制阐释”的全链条数据。中药数据具有典型的大数据特征，即“数据量大、种类丰富、复杂度高、数据价值高”。国际上，传统医药数据库大多从现代医学视角进行解读。ESCOP（欧洲草药治疗科学合作组织）数据库对草药临床应用、不良反应等数据进行了系统整理^［53］^。日本的 KNApSAcK（Kyoto Natural Products Screening Database）数据库专注于天然产物的代谢产物成分的收集，涵盖丰富的天然产物化学结构、生物活性及相关文献信息，包括63 715 条代谢产物和159 095 条代谢物-物种配对^［54］^。COCONUT 2.0（Collection of Open Natural Products）是目前最大的天然来源化学成分的免费数据库，收录了近70万条天然产物记录，具有高度的分子多样性和结构复杂性^［55］^。我国在中药数据库的建设方面起步较早，已建成多种特色鲜明的数据库。中国中医科学院中医药信息研究所自1984年起开展中医药学大型数据库建设（https://cintmed.cintcm.cn/cintmed/main.html），目前已构建48个数据库，数据总量达220余万条，打造了包含中医药期刊文献、各类中药、方剂等数据库在内的庞大体系。由西北农林科技大学等单位构建的TCMSP（Traditional Chinese Medicine Systems Pharmacology Database and Analysis Platform）数据库整合了中药化学成分、相关靶点及其药理作用等数据，为揭示中药作用机制、开发新药提供有力支持^［56］^。北京中医药大学等团队研发的SymMap数据库从分子机制和症状映射两个维度，将传统中医药与现代医学进行关联整合，提供草药/成分、靶点以及所治疗的临床症状和疾病等大量信息^［57］^。由北京中医药大学主导研发的HERB（本草组鉴）是一个基于现代医学标准构建的中医药数据库，包含了中医临床试验、高通量实验数据、相关参考文献等，并提供自动分析界面和知识图谱，支持中医药指导现代药物发现^［58］^。这些数据库从不同角度推动了中医药基础研究、临床研究及产业应用，为中医药传承创新提供了重要的数据保障。

然而，现有的中医药数据库的局限性也不容忽视，主要体现在：信息来源多而分散，质量参差不齐；数据整合、更新与维护不及时，数据共享机制不完善；中药特色挖掘不足，数据开发利用程度不足等^［59］^。北京大学陈语谦团队基于AI构建了世界最大中医药数据库TCMBank，提供了9 192种草药，61 966种无重复的成分，15 179个靶标，32 529种疾病及其成对关系，解决了中医药数据碎片化难题；此外，针对中西药之间可能发生的不良反应，设计了新型算法和模型来精准预测化合物之间的相互作用^［60］^。为应对上述挑战，AI在中药领域有着广阔的应用前景，在知识和数据采集挖掘、数据分析、化学成分分析、药理研究、临床应用和质量控制等多个领域形成专业数据库^［59，61，62］^。AI不仅突破了中药大数据研究的技术壁垒，更直接改变了中药这一传统学科的研究范式^［63］^。大连化物所团队一直致力于本草物质科学研究，现已全面开展大数据和AI学科布局。本研究团队以“药-靶-病”为核心逻辑主线，深度融合中医药特色数据（如中药方剂、药材/饮片、组分等）与现代生命科学和AI技术，在中药现代化研究领域已形成中药靶向药效物质基础和整合药理学研究的独特优势。依托中药大科学装置生成的大量数据，根据中药的“多组成、多靶点”特色，本研究团队正在建设高质量的“多靶点中药新药创制基础数据集”，将为中药领域提供TB量级的覆盖化学成分、结构解析与生物活性的标准化科学数据，和百万量级的专业文献分析与汇总数据。构建该基础数据集，不仅是为了整合分散的中药数据、提供可靠的数据资产，更是为了深度挖掘传统医药的价值，使其能为应对复杂疾病和全球健康挑战贡献独特智慧。

中药的物质组成结构多样，如生物碱、黄酮类等，且受药材来源与炮制工艺影响，呈现高度复杂性。与此同时，人体生理病理系统涉及基因、蛋白及代谢网络的动态调控，疾病状态下靶点动态变化，个体差异显著。这种双重复杂性导致传统湿实验（如体外细胞实验或动物模型）难以全面模拟中药与人体的非线性相互作用。通过计算模拟结合AI算法，整合中药小分子与靶点蛋白结构，研究中药药效物质与人体靶点蛋白的结合机制，模拟复杂的生物学过程，发现新分子、新靶点和新机制^［7，10，64-67］^。AI算法（如深度学习等）保证了模拟精度与效率^［68，69］^。虚拟筛选（virtual screening）是基于小分子数据库开展的活性化合物筛选，根据预设条件，在海量小分子中快速区分具备目标性质和不具备目标性质的化合物结构（如结合目标靶点的潜力），显著降低需要实验筛选的化合物数目、实验周期和成本^［70，71］^。分子对接（molecular docking）通过模拟小分子与靶点蛋白的结合模式和强度，预测氢键、疏水作用等关键相互作用，其本质是分子间（即受体和配体）的识别过程，包括分子间的空间匹配和能量匹配^［72］^。分子动力学（molecular dynamics）以经典力学、量子力学和统计力学为基础，模拟分子体系的运动，是研究蛋白质等生物大分子动态行为的有力工具，不仅揭示靶点蛋白与小分子之间复杂的相互作用，还为动态环境下的药-靶结合机制提供动态构象和能量变化^［73，74］^。此外，基于机器学习模型，结合结构化学、药理/毒理数据和临床信息，AI算法能快速处理复杂多维的数据，因而在中药小分子成药性预测与毒副作用评估（ADMET）中展现出独特优势^［75，76］^。

大数据和AI算法辅助的计算模拟和预测技术（图5）与中药的“多组分、多靶点”特性及人体生理病理复杂性结合，不仅能克服传统湿实验的局限性，如实验周期长、投入高、生成数据有限等，而且正在重塑中药研究范式，推动从“经验依赖”向“数据驱动”，显著加速中药药效物质基础研究与新药研发进程^［77，78］^。

**图5:**
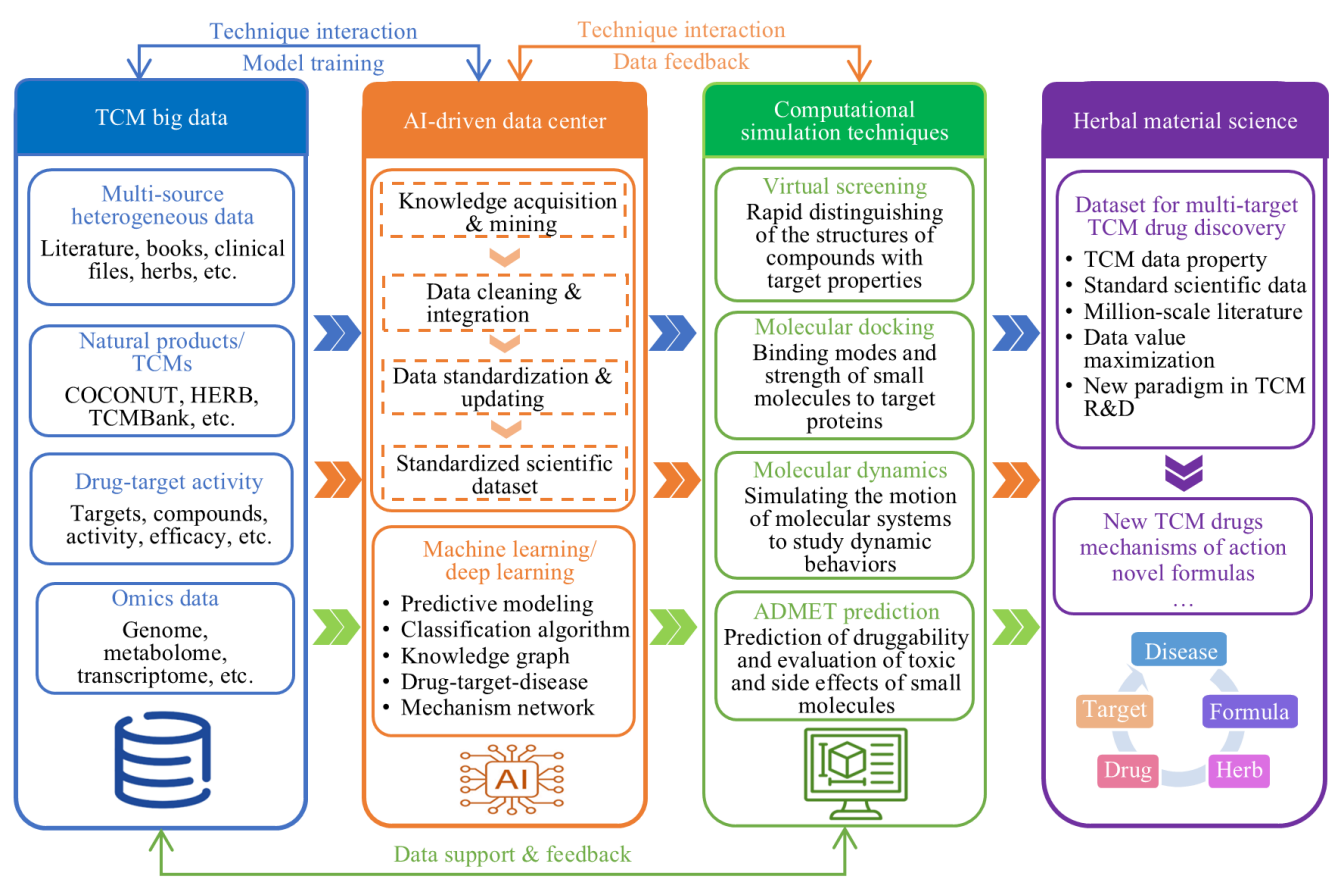
中药大数据与AI驱动的数据技术体系

### 3.3 结构生物学技术

蛋白质的结构与功能密切相关，特别是三维结构为理解生物学功能、疾病机制和药物研发提供了关键基础。中药药效成分的靶点与作用机制研究是本草物质科学研究的关键科学问题，更是中药现代化和国际化进程中的核心挑战^［79］^。中药活性分子通常通过与传统药物靶点（如GPCR、离子通道、激酶等）相互作用来发挥其多层面的生物学效应^［80］^。然而，该作用常呈现多靶点、多成分的复杂性，精确阐明其分子机制仍是该领域面临的巨大挑战。本草物质科学研究致力于中药的源头创新，即发现“新分子、新靶点、新机制”，为此，结构生物学技术被作为关键技术之一引入，旨在从原子分辨率水平上实现药物-靶标相互作用的可视化^［81，82］^。高分辨率的结构解析不仅为药物研发提供了精准的靶点信息，还可从原子层面揭示药物干预疾病的分子基础。例如，通过冷冻电镜解析中药葛根活性成分葛根素与脑内GABAA受体复合物的结构^［83］^，直接从原子层面揭示了其通过变构调节抑制神经通路从而减少脂肪吸收的全新机制。

结构生物学技术不仅解决了本草物质科学研究中传统经验与现代科学的衔接问题，也为阐释中药药效成分与靶点间的相互作用提供了结构范式。其中，3种主要技术得到广泛应用：X射线晶体学、核磁共振光谱学和冷冻电子显微镜。X射线晶体学是最为经典的技术，贡献了蛋白质数据库（PDB）中约90%的高分辨率结构，但其应用高度依赖于能否获得高质量三维晶体^［84］^。核磁共振光谱学的优势在于能在溶液环境中研究蛋白质的结构动态变化，适用于解析小分子配体的结合及蛋白质的柔性区域，但其应用范围受限于分子量（通常小于50 kDa）和谱图的复杂性^［85］^。相比之下，冷冻电子显微镜（Cryo-EM）技术因其近年来的“分辨率革命”而脱颖而出^［81，82］^。该技术无需结晶，通过将样品快速冷冻在玻璃态冰中直接观测，显著提高了蛋白结构解析的效率，尤其适用于难以结晶的膜蛋白和大型复合物的研究。随着直接电子探测器、自动化数据采集及三维重构算法的巨大进步，Cryo-EM的分辨率已常规达到原子水平（<2.5 Å），并且能够解析同一样品中不同构象状态的分布，从而揭示功能的动态过程。这些优势使其成为研究生物大分子复杂体系的前沿技术。Cryo-EM的技术特性与中药成分的多样性和作用模式的复杂性高度互补，为在结构层面系统阐释其生物学效应提供了前所未有的机遇。利用这些技术优势，开展中药高活性化合物-蛋白复合物的高分辨率三维结构研究，能够精准定位小分子的结合位点，并整合计算模拟及AI数据分析等技术，从原子水平阐明药物-靶标相互作用的动态过程。

中药中常存在大量结构相似而药效或毒性迥异的系列化合物（如同系物或衍生物），它们与同一靶点蛋白可形成一系列的药物-靶标复合物。利用Cryo-EM解析这一系列复合物的高分辨率结构，可通过精确对比，揭示官能团的细微差异如何影响其与结合口袋的相互作用，从而在原子水平上直接阐明构效关系（SAR），为高效、低毒的结构优化提供精准蓝图。更进一步，针对体现中药整体调节特点的多靶点作用机制，Cryo-EM能够解析同一活性成分与不同信号通路中的多个靶点蛋白形成的复合物结构。通过比较这些结构，可清晰揭示同一个分子如何以相同或不同的模式与多个靶点结合，从结构基础上解释其协同或拮抗效应，为多靶点药物的理性设计提供关键信息。最终，基于Cryo-EM获得的结构模型，可与分子对接、分子动力学模拟等计算生物学方法及点突变、细胞功能实验等生化手段形成强有力的交叉验证，推动中药活性成分研究从传统经验性描述迈向基于结构的精准、定量化时代，从而极大促进基于中药活性分子的创新药物研发，为中药现代化与国际化奠定坚实的科学基础。冷冻电镜在中药活性成分靶点验证与结构优化中的应用如图6所示。

**图6:**
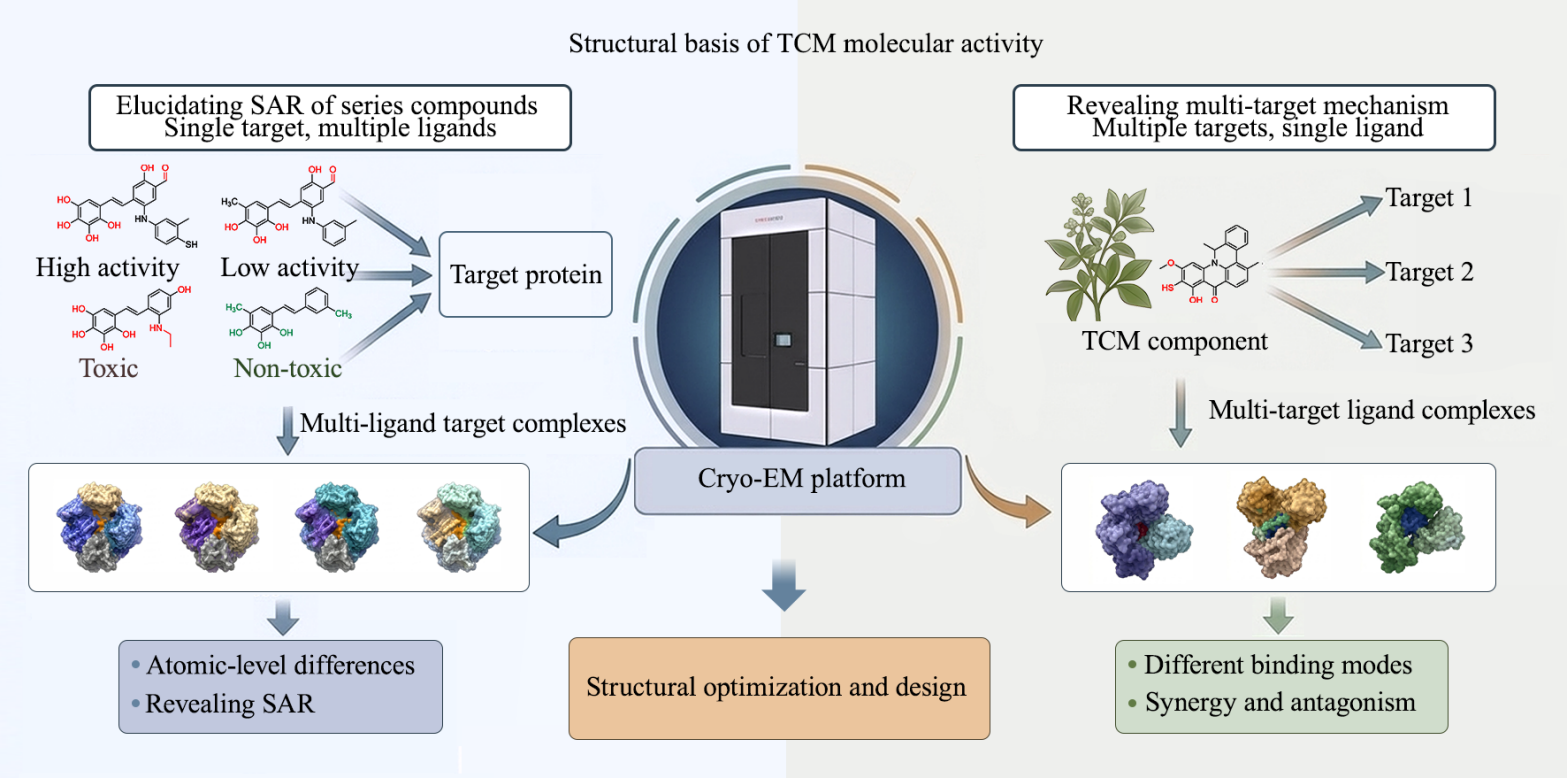
冷冻电镜用于中药活性成分靶点验证与结构优化研究

## 4 总结与展望

自本草物质组概念提出至今近20年来，分离纯化、液相色谱-质谱联用、核磁鉴定等分离分析技术显著进步，特别是多维多通道分离纯化预研装置的建成，突破了中药化合物样品规模化、系统化制备的瓶颈，极大提升了对中药物质组成结构的探测深度，为本草新化合物及新生物学效应的发现奠定了关键基础。与此同时，靶点活性发现、计算模拟与结构生物学等生物技术的快速发展，进一步推动了本草物质结构与功能之间关系的解析。随着相关技术的持续发展，本草物质科学研究将逐步揭示中药的未知领域，阐明多成分多靶点协同作用机制，并不断发掘新结构、新靶点及新生物学效应，为中药新药研发提供源头创新动力。

展望未来，本草物质科学研究方法与技术将继续沿着高通量、高分辨、智能化和多维融合的方向发展。随着多维高效制备色谱、在线/离线联用分离分析、智能质谱解析、AI辅助核磁鉴定、靶点发现技术、冷冻电镜及多组学计算模拟等技术的持续突破，本草物质研究将进一步实现从成分发现向结构解析、靶点确认、机制阐释及效应评价的系统性研究，并由经验驱动逐步转向数据与模型驱动。开展本草物质研究具有重要的科学意义：一方面，它是阐明中药药效物质基础、作用机制及安全性评价的前提；另一方面，它也是发现新结构、新靶点和新机制、推动新药研究的重要源头。更重要的是，本草物质科学研究直接关系到中药现代化的核心内涵，用科学语言和技术体系阐明中药科学原理，从而为中药标准化和现代化提供坚实的物质基础和方法学支撑。
